# Supplementary figures and images for: Y-Chromosome Haplotype Report among Eight Italian Horse Breeds
Source: Genes (Basel). 2023 Aug 9;14(8):1602. doi: 10.3390/genes14081602 (PMC10454838; doi:10.3390/genes14081602)

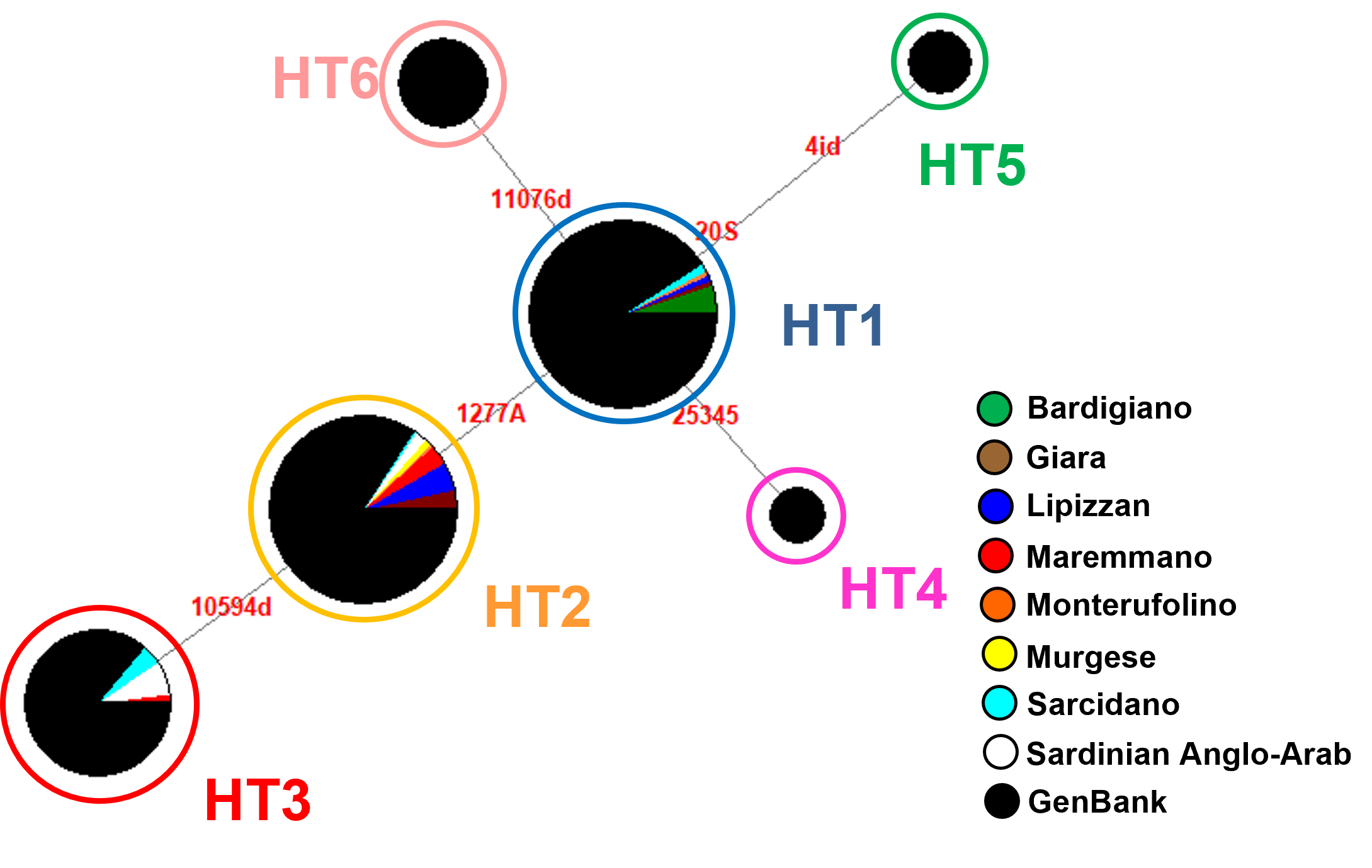

Supplement: Supplementary file 1 [file genes-14-01602-s001.zip › FigS1.png]
